# Clinical case and drug-drug interactions in the elderly: a case report

**DOI:** 10.1192/j.eurpsy.2026.10305

**Published:** 2026-07-31

**Authors:** G. Schoretsanitis

**Affiliations:** Unit of Pharmacogenetics and Clinical Psychopharmacology, Centre for Psychiatric Neuroscience, University of Lausanne, Lausanne, Switzerland

## Abstract

**Abstract:**

The management of elderly patients receiving polypharmacy represents a major interdisciplinary challenge in clinical routine due to various aspects, including the age-related physiological changes, the physical comorbidities, and the elevated for drug-drug interactions (DDIs). Rational psychopharmacology based on standardized assessment tools, including blood level assessments of medications, known as therapeutic drug monitoring (TDM), allow for treatment optimization in this highly vulnerable patient subgroup. Here we explicitly present the benefits of TDM assessments in the personalized management of elderly patients receiving polypharmacy. Such benefints include identification of DDIs, but also management of adherence issues and adverse drug reactions.

**Disclosure of Interest:**

G. Schoretsanitis Consultant of: consultant for Dexcel Pharma, HLS Therapeutics, Lundbeck and Thermo Fisher, Speakers bureau of: speaker’s fees from HLS Therapeutics, Lundbeck and, OM Suisse, Sadalax.

